# Abnormal brain functional and structural connectivity between the left supplementary motor area and inferior frontal gyrus in moyamoya disease

**DOI:** 10.1186/s12883-022-02705-2

**Published:** 2022-05-16

**Authors:** Junwen Hu, Yin Li, Zhaoqing Li, Jingyin Chen, Yang Cao, Duo Xu, Leilei Zheng, Ruiliang Bai, Lin Wang

**Affiliations:** 1 Department of Neurosurgery, the Second Affiliated Hospital, Zhejiang University School of Medicine, Jiefang Road 88th, Hangzhou, 310009 China; 2 Key Laboratory of Biomedical Engineering of Education Ministry, College of Biomedical Engineering and Instrument Science, Zhejiang University, 268 Kaixuan Road, South Central Building, Room 708, Hangzhou, 310027 Zhejiang China; 3 Department of Radiology, the Second Affiliated Hospital, Zhejiang University School of Medicine, Hangzhou, China; 4 Department of Psychiatry, the Second Affiliated Hospital, Zhejiang University School of Medicine, Hangzhou, China; 5 Department of Physical Medicine and Rehabilitation of the Affiliated Sir Run Run Shaw Hospital and Interdisciplinary Institute of Neuroscience and Technology, Zhejiang University School of Medicine, Hangzhou, China; 6 MOE Frontier Science Center for Brain Science and Brain-machine Integration, School of Brain Science and Brain Medicine, Zhejiang University, Hangzhou, China

**Keywords:** Moyamoya disease, Functional connectivity, White matter integrity, Cognitive function

## Abstract

**Background:**

Disruption of brain functional connectivity has been detected after stroke, but whether it also occurs in moyamoya disease (MMD) is unknown. Impaired functional connectivity is always correlated with abnormal white matter fibers. Herein, we used multimodal imaging techniques to explore the changes in brain functional and structural connectivity in MMD patients.

**Methods:**

We collected structural images, resting-state functional magnetic resonance imaging (rs-fMRI) and diffusion tensor imaging for each subject. Cognitive functions of MMD patients were evaluated using the Mini-Mental State Examination (MMSE), Montreal Cognitive Assessment (MoCA), and Trail Making Test parts A and B (TMT-A/-B). We calculated the functional connectivity for every paired region using 90 regions of interest from the Anatomical Automatic Labeling Atlas and then determined the differences between MMD patients and HCs. We extracted the functional connectivity of paired brain regions with significant differences between the two groups. Correlation analyses were then performed between the functional connectivity and variable cognitive functions. To explore whether the impaired functional connectivity and cognitive performances were attributed to the destruction of white matter fibers, we further analyzed fiber integrity using tractography between paired regions that were correlated with cognition.

**Results:**

There was lower functional connectivity in MMD patients as compared to HCs between the bilateral inferior frontal gyrus, between the bilateral supramarginal gyrus, between the left supplementary motor area (SMA) and the left orbital part of the inferior frontal gyrus (IFGorb), and between the left SMA and the left middle temporal gyrus (*P* < 0.01, FDR corrected). The decreased functional connectivity between the left SMA and the left IFGorb was significantly correlated with the MMSE (*r* = 0.52, *P* = 0.024), MoCA (*r* = 0.60, *P* = 0.006), and TMT-B (*r* = -0.54, *P* = 0.048) in MMD patients. White matter fibers were also injured between the SMA and IFGorb in the left hemisphere and were positively correlated with reduced functional connectivity.

**Conclusions:**

Brain functional and structural connectivity between the supplementary motor area and inferior frontal gyrus in the left hemisphere are damaged in MMD. These findings could be useful in the evaluation of disease progression and prognosis of MMD.

**Supplementary Information:**

The online version contains supplementary material available at 10.1186/s12883-022-02705-2.

## Background

Moyamoya disease (MMD) is a chronic cerebrovascular occlusion disease, of which the underlying pathogenesis has not yet been fully elucidated [1]. Chronic hypoperfusion in patients with MMD will inevitably damage white matter fibers [2]. The low perfusion or microstructurally altered tissue may appear normal on conventional magnetic resonance images, and are called normal-appearing white matter (NAWM) [3]. Aberrant neural functioning develops because of damage to white matter fibers or the neural pathway, which is linked to worse cognitive outcomes [4].

Resting-state functional magnetic resonance imaging (rs-fMRI) is widely used to study brain functional organization in patients with stroke and neurodegenerative diseases [5]. Functional connectivity is defined as the temporal correlation that equals a Pearson’s *r*, in the high amplitude, low-frequency spontaneously generated blood oxygen level-dependent (BOLD) signal between voxels or brain regions [6]. Exploring the characteristics of brain functional connectivity changes is beneficial because it can promote our knowledge regarding cognitive impairment secondary to chronic ischemia in MMD and is crucial for predicting the reversibility of neurocognitive dysfunctions after revascularization surgery. Thus, functional connectivity changes may become early features of cognitive impairment and a prognostic hallmark of MMD. Some researchers even suggest that rs-fMRI should be performed as part of routine clinical investigations that enable assessment of multiple brain systems in MMD [7].

Diffusion tensor imaging (DTI) has been used to quantify microstructural alterations in white matter in vivo, and is especially useful for patients with neurological disorders [8]. The main mechanism of DTI involves measuring the magnitude and directionality of random water movement to generate information regarding tissue or fiber integrity of the direct connections between different brain regions [9]. Fractional anisotropy (FA) and mean diffusivity (MD) are the primary tensor metrics that reflect the overall physiological and pathological alterations of white matter [10]. In addition, axial diffusivity (AD) and radial diffusivity (RD), which reflect axon integrity and myelin sheath integrity, respectively, can be useful for understanding the underlying physiology in many aspects [9, 11]. It has been confirmed that these DTI metrics are correlated to cognitive performances [12].

However, it remains unclear whether rs-fMRI can elucidate distinct brain functional characteristics using region-based analyses and whether the abnormal functional connectivity is related to altered cognition or white matter integrity in MMD. In the current study, we used rs-fMRI to explore the changes in brain functional connectivity in MMD patients on a large-scale level. Furthermore, DTI was used to study the subtle microstructural alterations of white matter fibers and their relationship to impaired brain functions in MMD patients. Our results could be applied as the hallmark in the evaluation of disease progression and prognosis of MMD.

## Methods

### Participants

The current study included 22 MMD patients and 10 healthy controls (HC). Patients were diagnosed with MMD based on digital subtraction angiography (DSA). Patients were right-handed and exhibited ischemia or hemorrhage with a time to onset of more than 3 months. The exclusion criteria were as follows: incomplete MRI data, revascularization surgery before the study, or cortical lesions on T1-weighted images (T1WI) larger than 3 mm. All imaging data were reviewed by two experienced clinicians (Dr. Xu and Dr. Wang). Patients were assessed for cognitive function by the same psychiatrist (Dr. Zheng) before the MRI scan. The cognitive assessment package included the Mini-Mental State Examination (MMSE), the Montreal Cognitive Assessment (MoCA), and the Trail Making Test parts A and B (TMT-A/-B). The total times (in seconds) for the TMT-A and TMT-B were used to represent the direct scores [13].

In addition, another 10 healthy participants that were gender-, age-, and education-matched with the MMD group were recruited as the control group. There were no brain lesions and no history of neurologic or systemic diseases in the participants in the control group. Our study was approved by the Human Research Ethics Committee of the Second Affiliated Hospital of Zhejiang University (ID: 2020–064), and written informed consent was obtained from each participant.

### MR imaging acquisition

The acquisition of study data was completed on a 3T MR scanner (Discovery MR750, GE) with an 8-channel head coil. The protocol included obtaining three-dimensional (3D) T1WI, rs-fMRI, and DTI. Spongy padding and earplugs were used by patients to decrease noise and head motion.

T1WIs were collected through 3D fast spoiled gradient-echo (3D-FSPGR): repetition time (TR) = 7.4 ms, echo time (TE) = 3.1 ms, flip angle = 8°, field of view (FOV) = 256 × 256 mm, slices = 170, and voxel size = 0.8 × 0.8 × 1.0 mm^3^. T2 fluid-attenuated inversion recovery (FLAIR) images were acquired with following parameters: TR = 8,400 ms, TE = 145.3 ms, flip angle = 90°, FOV = 512 × 512 mm, slices = 40, and voxel size = 0.39 × 0.39 × 4.0 mm^3^.

The diffusion-weighted images were acquired using 60 motion-probing gradient directions with *b*-values of 2,000 s/mm^2^ and two repetitions on *b* = 0 s/mm^2^, TR = 9,000 ms, TE = 92.7 ms, flip angle = 90°, FOV = 256 × 256 mm, slices = 68, and voxel size = 1.0 × 1.0 × 2.0 mm^3^. To correct the image distortion, we also obtained reverse phase-coded images.

The rs-fMRI was performed using a gradient echo planar imaging sequence with the following parameters: TR = 2,002 ms, TE = 30 ms, flip angle = 77°, FOV = 64 × 64 mm, slices = 38, voxel size = 3.44 × 3.44 × 4.0 mm^3^, and 180 continuous functional volumes were axially acquired.

### Rs-fMRI data analyses

#### Rs-fMRI data preprocessing

The functional images were preprocessed and analyzed using CONN toolbox v.20.b [14] based on Matlab (v.R2020b, MathWorks Inc., Sherborn, MA, USA). The first ten images were removed to avoid unstable magnetic artifacts. Then performing slice-timing correction to align rs-fMRI images to the center of the image. The remaining images were spatially realigned to the first image for head-motion correction. The mean realigned functional image was normalized to the EPI template in the Montreal Neurological Institute (MNI) space with a voxel size of 3 × 3 × 3 mm^3^ voxels. The data were smoothed with an 8-mm full width at half-maximum Gaussian kernel. We followed the default CONN denoising step that using a combination of aCompCor strategy (including white matter and cerebrospinal fluid signal, 5 components each), motion regression (12 regressors: 3 translation and 3 rotation + 6 first-order temporal derivatives), and scrubbing (74 noise components were identified) [15]. Then, the temporal signals in the 4-dimensional volume were linearly detrended and band-pass filtered (0.008–0.09 Hz) to remove undesired components.

#### Region of interest (ROI)-to-ROI connectivity analyses

The ROI-to-ROI analyses were performed in CONN toolbox by computing the temporal correlations between the BOLD signals from a given ROI with all other ROIs in the brain. Ninety ROIs without a cerebellum atlas were placed using the first version of the Anatomical Automatic Labeling atlas (AAL1). For each participant, Pearson correlations for all time-course pairs were computed and transformed into z-scores through Fisher’s transformation.

Group-level analysis was performed to compare differences in functional connectivity between MMD patients and HCs. A significant connection threshold was set to *P* < 0.01 and was false discovery rate (FDR) corrected. Additionally, the cluster threshold was set to *P* < 0.05, and was family-wise error (FWE) corrected.

### Post-hoc analyses

Functional connectivity values with significant differences in the ROI-to-ROI analyses between two groups were extracted in Matlab to perform a post-hoc correlation analysis with cognitive functions. The paired brain regions in which the functional connectivity was significantly correlated with cognitive function were further performing tractography to observe whether their structural connections were impaired according to tensor metrics.

#### Tractography

The tractography was performed in DSI studio (http://dsi-studio.labsolver.org/) with its default pipeline. The accuracy of the *b*-table orientation was examined by comparing fiber orientations with those of a population-averaged template [16]. A deterministic fiber tracking algorithm was used [17]. Two ROIs were placed at the left SMA and the left IFGorb, which were registered to each subject using the Advanced Normalization Tools (ANTs) (http://stnava.github.io/ANTs/) through default parameters. A seeding region was placed at the whole brain. The anisotropy threshold was 0.15, and the angular threshold was selected from a range of 15 degrees to 90 degrees. Tracks with a length shorter than 20 mm or longer than 100 mm were discarded. Trackings were terminated when a total of ten million seeds were placed. Finally, the tract statistics were extracted from DSI studio, including tract number and length, as well as tensor metrics.

### Statistical analyses

Statistical evaluations were performed using GraphPad Prism v8.0 (GraphPad Software, San Diego, CA, USA). The Mann–Whitney U tests were performed to determine group differences for clinical characteristics, functional connectivity, and tensor metrics. Moreover, Pearson’s correlation analyses were conducted to test whether the functional connectivity was related to the cognitive performances of MMD patients and tensor metrics. *P* < 0.05 was considered statistically significant.

## Results

### Clinical characteristics

A total of 22 MMD patients were included, with an average age of 46.8 ± 8.9 years and a gender ratio of 3:19 (men to women). 12 patients were ischemic MMD with symptoms including transient ischemic attack (*n* = 6, 27.3%), cerebral ischemia (*n* = 1, 4.5%), dizziness (*n* = 4, 18.2%) and headache (*n* = 1, 4.5%). 10 patients were hemorrhagic MMD with the most frequent manifestation was primary intraventricular hemorrhage (IVH) (*n* = 5, 22.7%), followed by IVH with intracerebral hemorrhage (ICH) (*n* = 2, 9.1%), IVH with subarachnoid hemorrhage (*n* = 1, 4.5%), and ICH only (*n* = 2, 23.9%). The symptomatic affected hemispheres comprised 10 left and 7 right, and 5 had no laterality of hemorrhage or ischemia. The angiographic results of Suzuki stages were as follows: stage I in 4 (18.2%), stage II in 7 (31.8%), stage III in 5 (22.7%), stage IV in 3 (13.6%), and stage V in 3 (13.6%) (Supplemental Table 1). There was no statistical difference in age, gender, or education between the MMD and HC groups. Cognitive performances of MMD patients were also demonstrated (Table 1).

**Table 1:** Baseline characteristics between MMD patients and HCs

	MMD (*n* = 22)	HC (*n* = 10)	*P* value
**Age (y) (mean ± SD)**	46.8 ± 8.9	49.7 ± 5.4	0.638
**Gender (men/women)**	3/19	2/8	0.506
**Education (y)**	8.7 ± 2.9	9.0 ± 3.7	0.352
**MMSE (mean ± SD)**	24.9 ± 4.1	/	/
**MoCA**	20.2 ± 6.5	/	/
**Working memory**	3.4 ± 1.4	/	/
**TMT-A (s)**	97.5 ± 46.0	/	/
**TMT-B (s)**	191.7 ± 89.6	/	/

Due to data loss in the early collection stage of this study, there are nineteen MMD patients in MMSE, MoCA, and working memory, and fourteen MMD patients in TMT-A and TMT-B

*MMD* Moyamoya Disease, *HC* Healthy Controls, *MMSE* Mini–Mental State Examination, *MoCA* Montreal Cognitive Assessment, *TMT-A/-B* Trail Making Test parts A and B

### Functional connectivity analyses

**Fig. 1:**
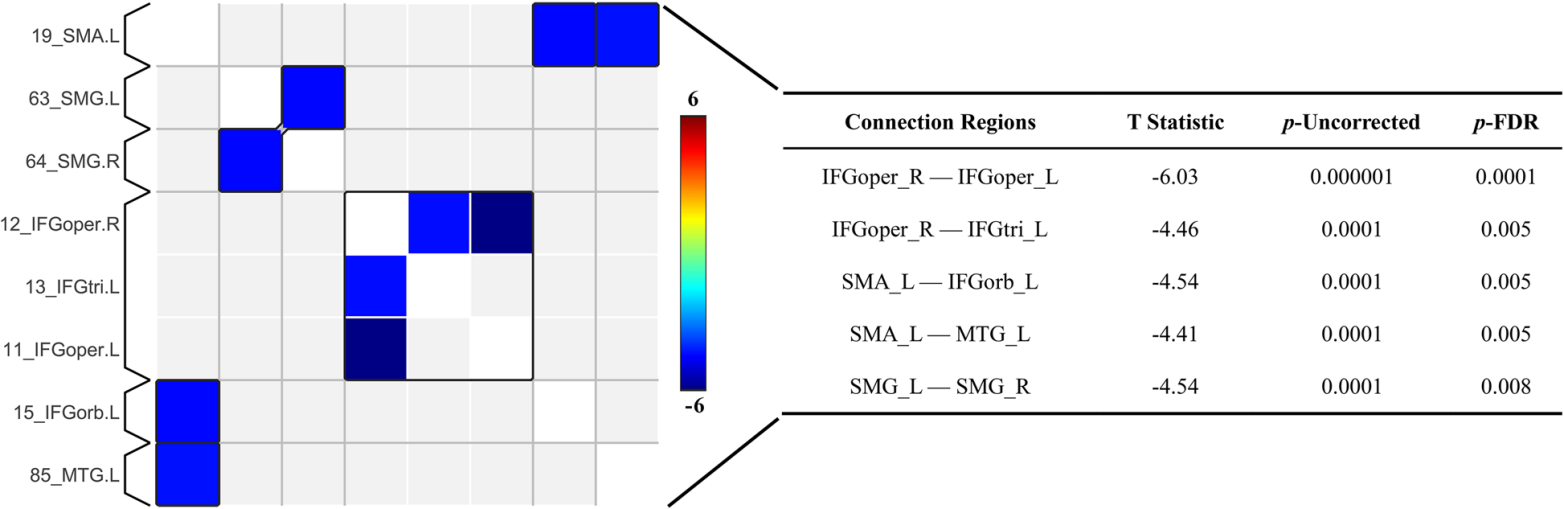
On the left, there is a connection matrix of the paired brain regions for MMD with significant differences after adjustment compared to healthy controls (*P* < 0.01, FDR corrected, FWE correction at *P* < 0.05). Blue regions represent reduced functional connectivity in patients with MMD. The table on the right lists the results of the comparison between the two groups. Abbreviations: SMA = supplementary motor area, SMG = supramarginal gyrus, IFGoper = opercular part of the inferior frontal gyrus, IFGtri = triangular part of the inferior frontal gyrus, IFGorb = orbital part of the inferior frontal gyrus, MTG = middle temporal gyrus; L denotes left, R denotes right

**Fig. 2:**
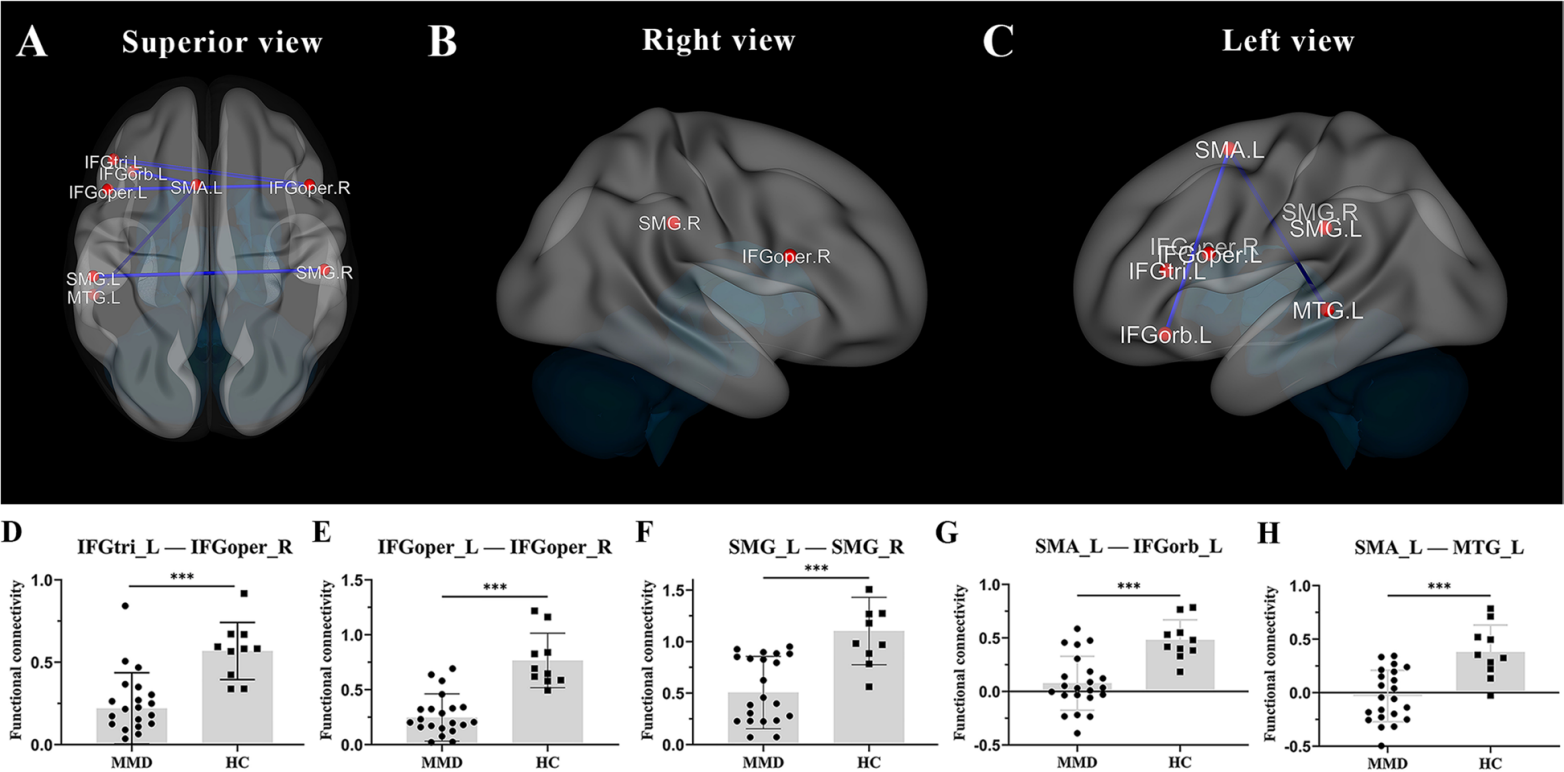
**A-C** The connection of brain regions with significant functional connectivity differences (blue lines) between patients with MMD and HCs are shown using three-dimensional images with superior, right, and left views, respectively. **D-H** Comparisons between the extracted functional connectivity values between the two groups. The red dots indicate where brain regions are located. Abbreviations: MMD = moyamoya disease, HC = healthy controls, SMA = supplementary motor area, SMG = supramarginal gyrus, IFGoper = opercular part of the inferior frontal gyrus, IFGtri = triangular part of the inferior frontal gyrus, IFGorb = orbital part of the inferior frontal gyrus, MTG = middle temporal gyrus; L denotes left, R denotes right. ^***^ *P* < 0.001

MMD and HC groups had different functional connectivity (Supplemental Fig. 1). MMD patients exhibited significant lower functional connectivity than HCs between the bilateral opercular part of the inferior frontal gyrus (IFGoper), between the right IFGoper and the left triangular part of the inferior frontal gyrus (IFGtri), between the left supplementary motor area (SMA) and the left orbital part of the inferior frontal gyrus (IFGorb), between the left SMA and the left middle temporal gyrus (MTG), and between the bilateral supramarginal gyrus (SMG) (*P* < 0.01, FDR corrected, FWE correction at *P* < 0.05) (Fig. 1). When comparing the extracted functional connectivity values, significant differences persisted between the MMD group and HCs (*P* < 0.001) (Fig. 2).

### Decreased left SMA-IFGorb functional connectivity correlates with MMD cognitive performances

**Fig. 3:**
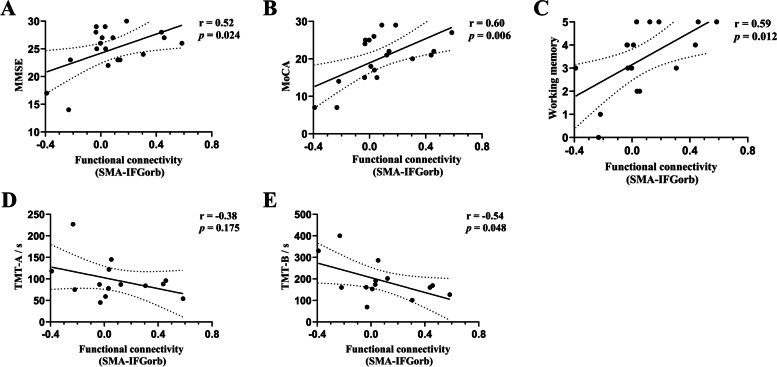
**A-E** Pearson’s correlation analyses were performed between the functional connectivity of the SMA-IFGorb in the left hemisphere and cognitive performances in MMD patients. The results show that the functional connectivity was significantly correlated with MMSE, MoCA, working memory, and TMT-B. Abbreviations: SMA = supplementary motor area, IFGorb = orbital part of the inferior frontal gyrus, MMSE = Mini–Mental State Examination, MoCA = Montreal Cognitive Assessment, TMT-A/-B = Trail Making Test parts A and B

Correlation analyses revealed that the functional connectivity between the bilateral IFGoper, between the right IFGoper and the left IFGtri, between the left SMA and the left MTG, and between the bilateral SMG were not correlated with MMD cognitive performances. However, the functional pathway of the left SMA-IFGorb had a significant positive correlation with the MMSE (*r* = 0.52, *P* = 0.024) and MoCA (*r* = 0.60, *P* = 0.006), and a negative correlation with the TMT-B (*r* = -0.54, *P* = 0.048) (Fig. 3).

### White matter between the left SMA-IFGorb injury

**Fig. 4:**
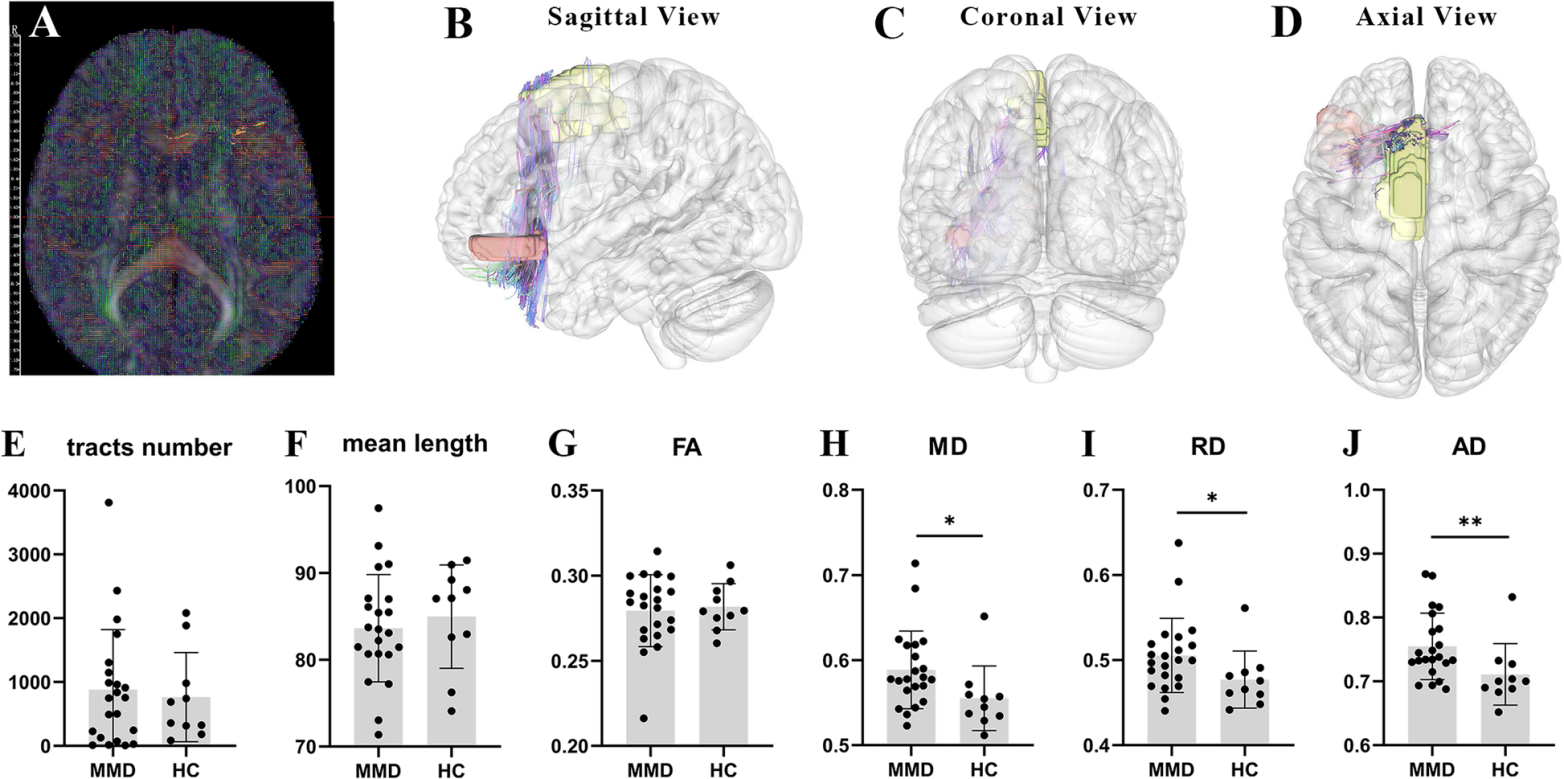
**A-D** These images of the brain illustrate a demonstration with one of the subjects. **A** The tensor map is shown, and **B-D** show the tractography results of the fibers between the left supplementary motor area (yellow block) and the orbital part of the inferior frontal gyrus (orange block). **E-J** Comparisons of the tract numbers, mean length, and tensor metrics of the fibers between the two groups. Abbreviations: MMD = moyamoya disease, HC = healthy controls, FA = fractional anisotropy, MD = mean diffusivity, RD = radial diffusivity, AD = axial diffusivity

In MMD patients, the number, average length, and FA of white matter fibers between SMA-IFGorb in the left hemisphere were not significantly different from those of the HCs, while MD (*P* < 0.05), RD (*P* < 0.05), and AD (*P* < 0.01) were significantly higher than those of HCs (Fig. 4). This suggests that the white matter fibers between SMA-IFGorb in the left hemisphere were extensively destroyed in MMD patients.

### Decreased functional connectivity correlates with impaired white matter fibers between left SMA-IFGorb

The functional connectivity between SMA-IFGorb in the left hemisphere was positively correlated with MD, RD, and AD of the fibers that connect the left SMA and IFGorb. The lower the functional connection values, the higher the MD, RD, and AD values (Supplemental Fig. 2).

## Discussion

Studying the functional organization of the brain in stroke and neurodegenerative diseases has sparked the interest of many researchers in recent years. Although there have been several studies applying functional MRI techniques in the realm of MMD to explore ‘small-world’ network topologies, dynamic functional connectivity networks, and modularity in MMD patients [7, 18, 19], the current study performed ROI-based analyses via functional connectivity for the first time and further combined tensor metrics to provide additional evidence for our findings.

There is growing evidence that brain functional changes may occur prior to the structural changes and imply a dissociation between functional impairment and structural damage [20, 21]. While the BOLD signal contrast depends on the interplay among hemodynamic factors [22], this may explain the physiology underlying reduced interhemispheric or intrahemispheric functional connectivity after stroke; thus, changes in brain function are likely to be more sensitive than that in structural pathways.

Our study showed that the functional connectivity of several paired regions decreased in MMD patients. There is increasing evidence that intrahemispheric or interhemispheric connectivity changes that occur in ischemic disease [23] are linked to cognitive decline [24]. Here, we also confirmed that changes in functional connectivity were correlated with cognitive performances in MMD, although significant correlation was only demonstrated in the functional connectivity between SMA-IFGorb in left hemisphere. The functional pathway of the left SMA-IFGorb had a significant positive correlation with the MMSE and MoCA, and a negative correlation with the TMT-B. Since the larger TMT-B scores indicate worse performance, it means that this pathway is positively correlated with general cognitive function and processing speed or set-shifting abilities.

The SMA complex is located in the posterior part of the superior frontal gyrus, which is bounded by the cingulate gyrus, anterior central gyrus, and superior frontal gyrus [25]. Neuroimaging studies have confirmed that the SMA is involved in the cognitive process of working memory, executive control and processing speed [26–28]. Through fiber tracking imaging, researchers found that the most important subcortical connection with the SMA included the primary motor area, the cingulate, the corpus callosum, the inferior frontal gyrus (IFG), and the striatal complex-internal capsule [29]. It is important to mention that the frontal aslant tract has been identified as a white matter tract connecting the SMA to the IFG, and it was shown to have a role in language function, working memory, social community tasks, attention, and processing [30]. Our results were consistent with the abovementioned anatomical and imaging studies of the SMA.

MMD is an ischemic disease that affects patients for a long period of time, and chronic insufficient blood supply leads to demyelination of white matter fibers and destruction of axon integrity. However, such patterns of subtle damage referred to as NAWM are usually not demonstrated in the structural images; thus, preventing us from detecting the brain abnormalities that are caused by MMD on conventional MRI sequences. Here, in this study, we excluded patients with cerebral lesions larger than 3 mm. In ischemic MMD with symptoms including TIA, dizziness, or headache, there is always no apparent cerebral lesion. In hemorrhagic MMD, cerebral lesions could be the residual minor destruction of brain structures after the absorption of hematoma. And especially in patients with primary IVH, there is always no evidence of lesions in the parenchyma. Fortunately, with the development of MR technology, we can use emerging methods to elucidate brain damage by capitalizing on many different aspects other than structure.

Currently, there is intense research being conducted on white matter integrity and brain functional connectivity in neurological diseases. Studies have shown that white matter abnormalities are correlated with disrupted functional connectivity, suggesting that common mechanisms may underlie fibers and functional connectivity [31]. Our study revealed that the fiber integrity and functional connectivity between the SMA and the IFGorb in the left hemisphere were disrupted before the development of a significant brain lesion in MMD patients. Such early detection of brain abnormalities at the preclinical stage is conducive for implementing preventive interventions to abate cognitive decline in MMD.

FA, MD, RD, and AD are the main tensor metrics that are derived from DTI. When the white matter fibers are damaged, the diffusion range of water molecules in the fibers increase, and the values of FA, MD, and RD will also increase accordingly. Our study showed that the FA of the fibers between the SMA and the IFGorb were not significantly different between MMD patients and HCs. This might be the result of the existence of cross fibers between the two brain regions, or it is highly likely that it occurred because the disruption of the fibers happened in all directions.

The interpretations of the changes in the AD are controversial. Theoretically, the AD values decrease when axons are injured, but other studies report that the AD increases in damaged white matter regions [32, 33]. The potential mechanism consists of increased extracellular water content that is secondary to atrophy of white matter fibers, and alterations in the water flow in the axons occur due to the breakdown of certain components of the cytoskeleton, which allows water molecules to move more rapidly parallel to the axons [32, 34]. To summarize, in the case of intensive axonal loss, the increased isotropic diffusion is likely to enhance both RD and AD [33].

Though it seems a bit of redundancy across the cognitive assessment package that we used, there still lies some differences between MMSE, MoCA, and TMT. For example, the MMSE is likely a better test for more severe cognitive impairment and MoCA for mild conditions [35]. TMT is also different from the block that is included in MoCA. Research suggested that TMT had a better performance than MoCA in some cases [36]. On the other hand, the TMT is a more precise indicator of reduced visual processing speed [37]. While they have different applications and results, combining these measurements may provide us with a more comprehensive understanding of cognitive impairment and recovery after revascularization surgery in MMD patients.

CONN is a Matlab-based software that has been widely used for the calculation, demonstration, and investigation of brain network connectivity. Connectivity assessment techniques include seed-to-voxel network maps, ROI-to-ROI connectivity matrixes, graph characteristics of network systems, brain interconnection, inherent connectivity, generalized psychophysiological interaction models, and other voxel-to-voxel measures. Unlike seed-based correlation analysis, which is a hypothesis-driven approach based on a chosen region, ROI-to-ROI analysis is a data-driven method that can produce information regarding the entire brain connectivity pattern. Therefore, we used ROI-based analyses in this study, which we considered to be more powerful for investigating the abnormal brain functional connectivity at the regional level.

White matter integrity impairment and cognitive disruption in MMD have been demonstrated in several studies, aberrant functional connectivity is another form of brain damage caused by MMD [2, 7, 18, 38]. The impaired brain functional and structural connectivity between the SMA and IFG in the left hemisphere provides us a novel perception of the pathophysiology of MMD and might be applied as the hallmark in the evaluation of disease progression and prognosis of MMD in the future.

There are some limitations to our study that must be mentioned. First, like other MMD imaging studies, a small number of patients were investigated due to the rare incidence of the disease, which limits the generalizability of our results. It is necessary to conduct a multi-center, large-sample study to confirm our results. Due to the impact of COVID-19, the collection of control cases has also been affected, though a matched study is more appropriate. Second, vascular abnormalities of MMD mainly involve the anterior circulation of the brain, and therefore, our study did not include the cerebellum as a ROI. It is worth noting that some studies indicate the existence of a functional connection between the cerebellum and the brain [5]. Third, anatomical and imaging studies have shown rich fiber connections between the SMA and the IFG that are associated with cognitive functioning, whereas our results show only that the breakdown of the connection between the SMA and the IFGorb in the left hemisphere is significantly correlated with the cognitive performance of MMD patients. Fourth, further studies are required to determine whether functional or structural connectivity is initially damaged before the onset of cognitive dysfunction in patients with MMD.

## Conclusion

In Moyamoya disease, there is damaged brain functional connectivity between the supplementary motor area and the inferior frontal gyrus in the left hemisphere, which is correlated with incomplete integrity of white matter fibers, and may contribute to impaired cognitive performance. These findings could be useful in the evaluation of disease progression and prognosis of moyamoya disease.

## Supplementary Information

**Additional file 1:** **Supplemental Figure 1.** This matrix is a summary of 90 ROIs from the Anatomical Automatic Labeling template-based statistical parametric maps of functional connectivity analyzed between the MMD group and HC. The matrix is 90 x 90, and each voxel stores a T statistic value ranging from 6 to -6. The red voxels indicate the functional connectivity of the MMD group is greater than HC, and blue is the opposite. The color bar is located at the right. **Supplemental Figure 2.** A-C The correlation between functional connectivity and tensor metrics of the SMA-IFGorb in the left hemisphere of MMD patients and HC. **Supplemental Table 1.** Demographics and clinical characteristics of 22 MMD patients.

**Additional file 2: Supplemental Table 2.** Regions of interest included in AAL-atlas.

## Data Availability

All data generated or analyzed during this study are included in this published article [and its supplementary information files].

## Abbreviations

MMD: Moyamoya disease
NAWM: Normal-appearing white matter
rs-fMRI: Resting-state functional magnetic resonance imaging
BOLD: Blood oxygen level-dependent
DTI: Diffusion tensor imaging
SMA: Supplementary motor area
IFGorb: Orbital part of the inferior frontal gyrus
FA: Fractional anisotropy
MD: Mean diffusivity
AD: Axial diffusivity
RD: Radial diffusivity
HC: Healthy controls
DSA: Digital subtraction angiography
MMSE: Mini-Mental State Examination
MoCA: Montreal Cognitive Assessment
TMT-A/-B: Trail Making Test parts A and B
