# Supplementary material for: Abnormal brain functional and structural connectivity between the left supplementary motor area and inferior frontal gyrus in moyamoya disease
Source: BMC Neurol. 2022 May 16;22:179. doi: 10.1186/s12883-022-02705-2 (PMC9108139; doi:10.1186/s12883-022-02705-2)
Supplement: Supplementary file 1 — Additional file 1: Supplemental Figure 1. This matrix is a summary of 90 ROIs from the Anatomical Automatic Labeling template-based statistical parametric maps of functional connectivity analyzed between the MMD group and HC. The matrix is 90 x 90, and each voxel stores a T statistic value ranging from 6 to -6. The red voxels indicate the functional connectivity of the MMD group is greater than HC, and blue is the opposite. The color bar is located at the right. Supplemental Figure 2. A-C The correlation between functional connectivity and tensor metrics of the SMA-IFGorb in the left hemisphere of MMD patients and HC. Supplemental Table 1. Demographics and clinical characteristics of 22 MMD patients. [file 12883_2022_2705_MOESM1_ESM.pdf]

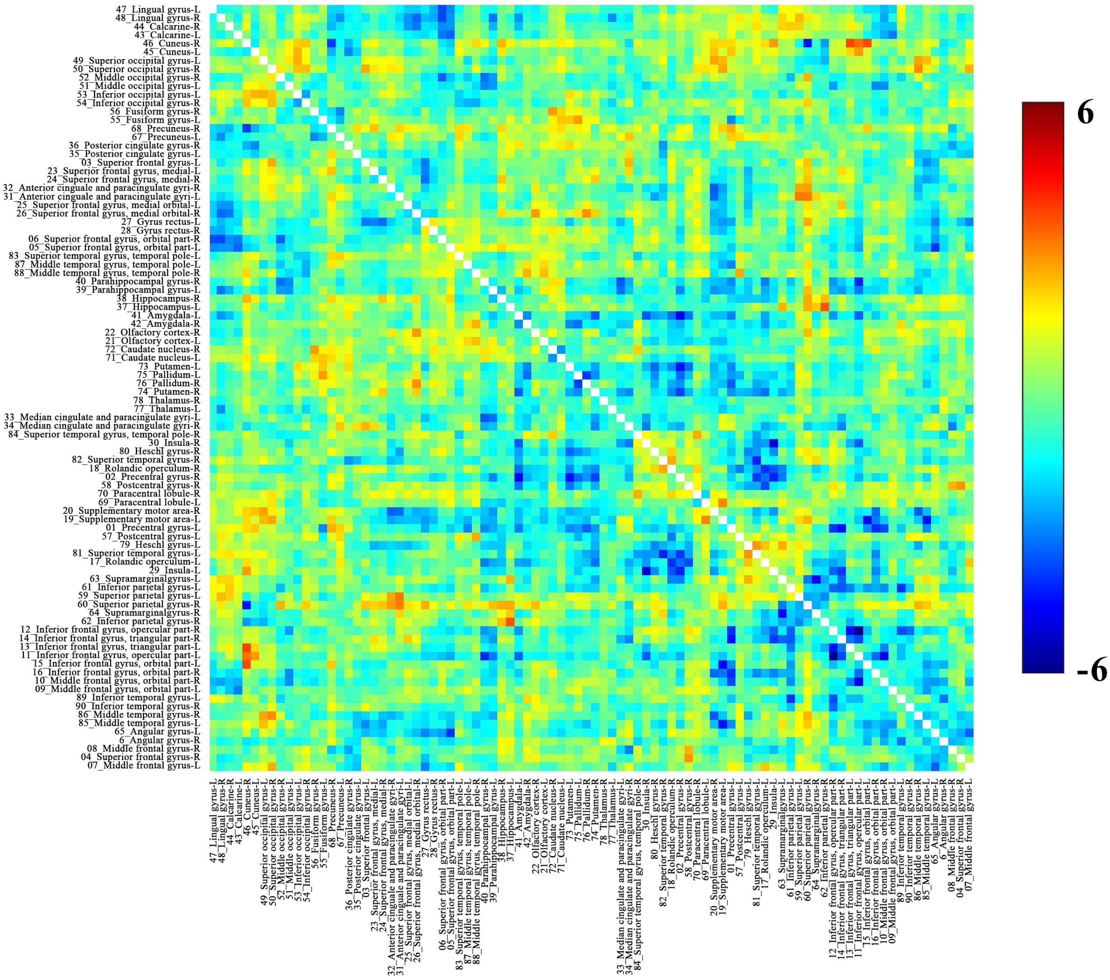

Supplemental Figure 1. This matrix is a summary of 90 ROIs from the Anatomical Automatic Labeling template-based statistical parametric maps of functional connectivity analyzed between the MMD group and HC. The matrix is 90 x 90, and each voxel stores a *T* statistic value ranging from 6 to -6. The red voxels indicate the functional connectivity of the MMD group is greater than HC, and blue is the opposite. The color bar is located at the right.

Abbreviation: MMD = moyamoya disease, HC = healthy controls, ROI = region of interest.

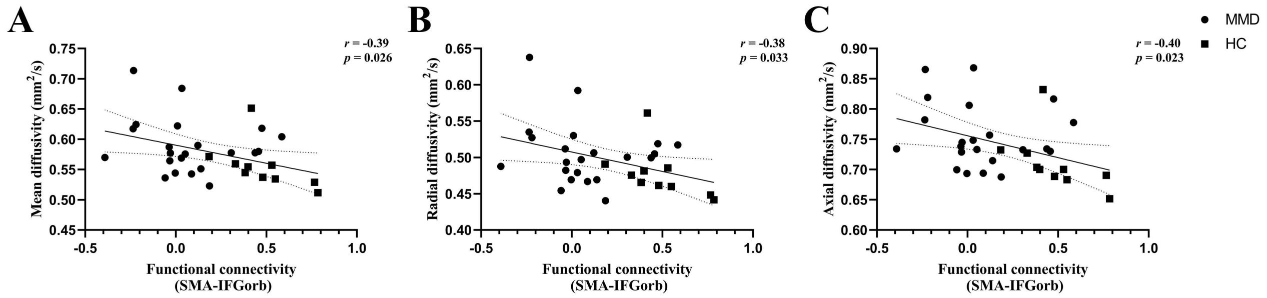

Supplemental Figure 2. A-C The correlation between functional connectivity and tensor metrics of the SMA-IFGorb in the left hemisphere of MMD patients and HC.

Abbreviations: MMD = moyamoya disease, HC = healthy controls, SMA = supplementary motor area, IFGorb = orbital part of the inferior frontal gyrus.

Supplemental Table 1. Demographics and clinical characteristics of 22 MMD patients

Subject	Age	Gender	Clinical manifestation	Mainly Affected Hemisphere	Time to onset	Smoking	Alcohol	HTN	Diabetes	HPL	Comorbidity	Suzuki stage	mRS grade
P001	51	F	Dizziness and headache	/	2 years	N	N	N	Y	N	Gastritis	V	1
P002	30	M	TIA	left	1 month	Y	N	N	N	N	Erosive gastritis	II	1
P003	45	M	TIA	left	1 month	Y	N	Y	N	N	Chronic colonitis	III	1
P004	44	F	IVH	right	3 months	N	N	N	N	Y	/	III	2
P005	34	F	IVH	left	3 months	N	N	N	N	Y	ACoA	I	0
P006	36	M	Thalamic hemorrhage with IVH	right	3 months	Y	N	N	N	N	/	II	0
P007	42	F	IVH	right	3 months	N	N	N	N	N	/	III	1
P008	44	F	Headache	left	1 year	N	N	N	N	Y	/	IV	1
P009	53	F	TIA	right	6 months	N	Y	Y	N	N	/	IV	1
P010	58	F	Dizziness	/	6 months	N	N	N	Y	N	/	II	1
P011	54	F	TIA	left	2 months	N	N	Y	Y	N	/	I	1
P012	45	F	IVH with subarachnoid hemorrhage	right	4 months	N	N	N	N	N	/	II	1
P013	56	F	Cerebral ischemia	left	5 months	N	N	N	N	N	/	V	1
P014	57	F	Dizziness	/	6 months	N	N	Y	Y	N	/	I	1
P015	34	F	Basal ganglia hemorrhage	left	18 months	N	N	N	N	N	/	III	1
P016	50	F	Basal ganglia hemorrhage with IVH	left	6 months	N	N	N	N	N	/	IV	1
P017	52	F	Thalamic hemorrhage	right	5 months	N	N	N	Y	N	/	II	1
P018	53	F	Dizziness	/	10 years	N	N	Y	Y	N	/	I	1
P019	52	F	TIA	left	6 months	N	N	N	N	N	Cervicitis	V	1
P020	56	F	IVH	/	4 months	N	N	N	N	N	Spinal disc herniation	III	0
P021	31	F	TIA	left	2 months	N	N	N	N	N	/	II	1
P022	53	F	IVH	right	3 months	N	N	N	N	N	Lung nodules	II	0

Note. HTN = hypertension; HPL = hyperlipidemia; mRS = modified Rankin Scale; TIA = transient ischemic attacks; IVH = intraventricular hemorrhage; ACoA = anterior communicating aneurysm.

'/' in Mainly Affected Hemisphere means there is no laterality of hemorrhage or ischemia.
