# Supplementary material for: Abnormal brain functional and structural connectivity between the left supplementary motor area and inferior frontal gyrus in moyamoya disease
Source: BMC Neurol. 2022 May 16;22:179. doi: 10.1186/s12883-022-02705-2 (PMC9108139; doi:10.1186/s12883-022-02705-2)
Supplement: Supplementary file 2 — Additional file 2: Supplemental Table 2. Regions of interest included in AAL-atlas. [file 12883_2022_2705_MOESM2_ESM.pdf]

Supplemental Table 2. Regions of interest included in AAL-atlas

Labels	abbr.	Regions	Labels	abbr.	Regions
1	PreCG.L	Precental gyrus	46	CUN.R	Cuneus
2	PreCG.R	Precental gyrus	47	LING.L	Lingual gyrus
3	SFG.L	Superior frontal gyrus	48	LING.R	Lingual gyrus
4	SFG.R	Superior frontal gyrus	49	SOG.L	Superior occipital gyrus
5	SFGorb.L	Superior frontal gyrus, orbital part	50	SOG.R	Superior occipital gyrus
6	SFGorb.R	Superior frontal gyrus, orbital part	51	MOG.L	Middle occipital gyrus
7	MFG.L	Middle frontal gyrus	52	MOG.R	Middle occipital gyrus
8	MFG.R	Middle frontal gyrus	53	IOG.L	Inferior occipital gyrus
9	MFGorb.L	Middle frontal gyrus, orbital part	54	IOG.R	Inferior occipital gyrus
10	MFGorb.R	Middle frontal gyrus, orbital part	55	FG.L	Fusiform gyrus
11	IFGoper.L	Inferior frontal gyrus, opercular part	56	FG.R	Fusiform gyrus
12	IFGoper.R	Inferior frontal gyrus, opercular part	57	PoCG.L	Postcentral gyrus
13	IFGtri.L	Inferior frontal gyrus, triangular part	58	PoCG.R	Postcentral gyrus
14	IFGtri.R	Inferior frontal gyrus, triangular part	59	SPG.L	Superior parietal gyrus
15	IFGorb.L	Inferior frontal gyrus, orbital part	60	SPG.R	Superior parietal gyrus
16	IFGorb.R	Inferior frontal gyrus, orbital part	61	IPG.L	Inferior parietal gyrus
17	ROL.L	Rolandic operculum	62	IPG.R	Inferior parietal gyrus
18	ROL.R	Rolandic operculum	63	SMG.L	Supramarginal gyrus
19	SMA.L	Supplementary motor area	64	SMG.R	Supramarginal gyrus
20	SMA.R	Supplementary motor area	65	ANG.L	Angular gyrus
21	OLF.L	Olfactory cortex	66	ANG.R	Angular gyrus
22	OLF.R	Olfactory cortex	67	PCUN.L	Precuneus
23	SFGmed.L	Superior frontal gyrus, medial	68	PCUN.R	Precuneus
24	SFGmed.R	Superior frontal gyrus, medial	69	PCL.L	Paracentral lobule
25	SFGmorb.L	Superior frontal gyrus, medial orbital	70	PCL.R	Paracentral lobule
26	SFGmorb.R	Superior frontal gyrus, medial orbital	71	CAU.L	Caudate nucleus
27	REC.L	Gyrus rectus	72	CAU.R	Caudate nucleus
28	REC.R	Gyrus rectus	73	PUT.L	Lenticular nucleus, putamen
29	INS.L	Insula	74	PUT.R	Lenticular nucleus, putamen
30	INS.R	Insula	75	PAL.L	Lenticular nucleus, pallidum
31	ACG.L	Anterior cingulate and paracingulate gyri	76	PAL.R	Lenticular nucleus, pallidum
32	ACG.R	Anterior cingulate and paracingulate gyri	77	THA.L	Thalamus
33	MCG.L	Median cingulate and paracingulate gyri	78	THA.R	Thalamus
34	MCG.R	Median cingulate and paracingulate gyri	79	HES.L	Heschl gyrus
35	PCG.L	Posterior cingulate gyrus	80	HES.R	Heschl gyrus
36	PCG.R	Posterior cingulate gyrus	81	STG.L	Superior temporal gyrus
37	HIP.L	Hippocampus	82	STG.R	Superior temporal gyrus
38	HIP.R	Hippocampus	83	STGp.L	Superior temporal gyrus: temporal pole
39	PHIP.L	Parahippocampal gyrus	84	STGp.R	Superior temporal gyrus: temporal pole
40	PHIP.R	Parahippocampal gyrus	85	MTG.L	Middle temporal gyrus
41	AMYG.L	Amygdala	86	MTG.R	Middle temporal gyrus
42	AMYG.R	Amygdala	87	MTGp.L	Middle temporal gyrus: temporal pole
43	CAL.L	Calcarine fissure and surrounding cortex	88	MTGp.R	Middle temporal gyrus: temporal pole
44	CAL.R	Calcarine fissure and surrounding cortex	89	ITG.L	Inferior temporal gyrus
45	CUN.L	Cuneus	90	ITG.R	Inferior temporal gyrus
